# Single Dose of a Small Molecule Leads to Complete Regressions of Large Breast Tumors in Mice

**DOI:** 10.1021/acscentsci.4c01628

**Published:** 2025-01-22

**Authors:** Michael P. Mulligan, Matthew W. Boudreau, Brooke A. Bouwens, Yoongyeong Lee, Hunter W. Carrell, Junyao Zhu, Spyro Mousses, David J. Shapiro, Erik R. Nelson, Timothy M. Fan, Paul J. Hergenrother

**Affiliations:** † Department of Chemistry, University of Illinois at Urbana-Champaign, Urbana, Illinois 61801, United States; ‡ Carl R. Woese Institute for Genomic Biology, University of Illinois at Urbana-Champaign, Urbana, Illinois 61801, United States; § Department of Biochemistry, University of Illinois at Urbana-Champaign, Urbana, Illinois 61801, United States; ∥ Department of Comparative Biosciences, University of Illinois at Urbana-Champaign, Urbana, Illinois 61802, United States; ⊥ Systems Oncology, Scottsdale, Arizona 85255, United States; # Cancer Center at Illinois, University of Illinois at Urbana-Champaign, Urbana, Illinois 61801, United States; 7 Department of Molecular and Integrative Physiology, University of Illinois at Urbana-Champaign, Urbana, Illinois 61801, United States; 8 Division of Nutritional Sciences, University of Illinois at Urbana-Champaign, Urbana, Illinois 61801, United States; 9 Beckman Institute for Advanced Science and Technology, University of Illinois at Urbana-Champaign, Urbana, Illinois 61801, United States; 10 Department of Veterinary Clinical Medicine, University of Illinois at Urbana-Champaign, Urbana, Illinois 61802, United States

## Abstract

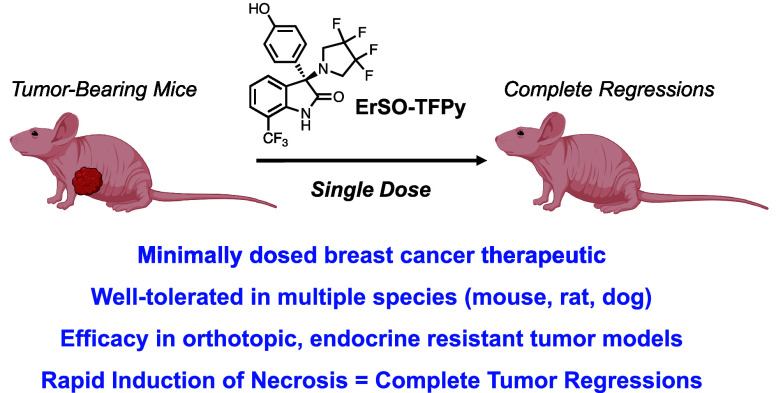

Patients with estrogen receptor α positive (ERα+) breast cancer typically undergo surgical resection, followed by 5–10 years of treatment with adjuvant endocrine therapy. This prolonged intervention is associated with a host of undesired side effects that reduce patient compliance, and ultimately therapeutic resistance and disease relapse/progression are common. An ideal anticancer therapy would be effective against recurrent and refractory disease with minimal dosing; however, there is little precedent for marked tumor regression with a single dose of a small molecule therapeutic. Herein we report **ErSO-TFPy** as a small molecule that induces quantitative or near-quantitative regression of tumors in multiple mouse models of breast cancer with a single dose. Importantly, this effect is robust and independent of tumor size with eradication of even very large tumors (500−1500 mm^3^) observed. Mechanistically, these tumor regressions are a consequence of rapid induction of necrotic cell death in the tumor and are immune cell independent. If successfully translated to human cancer patients, the benefits of such an anticancer drug that is effective with a single dose would be significant.

## Abstract 2

A single dose of **ErSO-TFPy** leads to complete tumor regressions in multiple mouse xenograft models of breast cancer.

## Introduction

Breast cancer is the second leading cause of cancer-related mortality in women, with annual cases in the US exceeding 250,000, leading to >40,000 deaths per year.^1^ The majority of breast cancers (∼70%) are estrogen receptor alpha positive (ERα+), with activation by estrogen promoting survival and growth of breast cancer cells through the activity of ERα.^2,3^ In combination with surgery, endocrine therapies targeting estrogen synthesis (e.g., aromatase inhibitors) or ERα-mediated transcriptional activity (e.g., tamoxifen, fulvestrant, elacestrant) are mainstay treatments for ERα+ breast cancer and lead to better outcomes and are better tolerated than chemotherapy.^4,5^ However, there remains a critical need for new treatments for late-stage breast cancer, where the median survival is three years.^6^

In the adjuvant setting where endocrine therapy is most successful, patients are required to take daily oral medications of an aromatase inhibitor or tamoxifen for 5–10 years following surgical resection.^7^ While this treatment regimen improves outcomes for breast cancer, it is associated with increased incidence of endometrial cancer, pulmonary embolisms, and osteoporosis.^8,9^ Long-term endocrine therapy is also associated with other side effects such as thromboembolic events, musculoskeletal pain, sexual dysfunction, and fatigue that decrease patient quality of life, exacerbated in premenopausal women where ovarian function suppression is common.^7^ These side effects from endocrine therapy lessen patient compliance and adherence to therapeutic regimens, with an estimated 20−30% of patients ultimately discontinuing treatment, thus reducing the efficacy of these therapies.^10,11^

Unfortunately, even with these long-term treatments for ERα+ breast cancer, the probability of recurrence remains static beyond five years following diagnosis, and consequently, many patients (30–50%) receiving prolonged endocrine therapy will still progress to advanced breast cancer that is incurable with current treatments. Resistance can occur through many mechanisms including the direct mutation of ERα (with the D538G and Y537S variants being the most common), leading to ligand-independent activation, and through activation of pathways proximal to ERα such as CDK4/6 signaling and PI3K/AKT/mTOR signaling.^4^ Such resistance occurs partly because endocrine therapies typically are cytostatic: tumor cell proliferation is inhibited, but cell death is modest.^12,13^ Consistent with this cytostatic activity, in preclinical mouse models many ERα-targeted therapies (including state-of-the-art combination therapy^14−16^) retard tumor growth but fail to induce regression. Breast cancer patients would derive the greatest benefit from an anticancer drug that potently and selectively kills cancer cells and requires dosing only a few times (or once) to exert its effect; such limited dosing could thwart resistance, prevent the development of secondary cancers, and reduce side effects. Of course, other than some immunotherapies,^17,18^ dramatic efficacy with minimal dosing has little precedent in anticancer therapy.

We recently reported the discovery of **ErSO**, a small molecule that dysregulates cation homeostasis in ERα+ breast cancer cells leading to cell swelling and rapid necrotic death in sensitive cell lines.^19,20^ Assessment in multiple mouse models of ERα+ breast cancer showed that **ErSO** and the next-generation derivative **ErSO-DFP**, given daily or weekly, produce impressive tumor regressions and in some cases lead to complete tumor eradication.^19,21^ Here we report the evaluation of **ErSO-TFPy**, a compound with enhanced anticancer potency and selectivity, in multiple challenging human tumor models implanted into mice. Unlike **ErSO**, **ErSO-TFPy** is tolerated in rodents at high intravenous (IV) doses. This trait, combined with rapid induction of cell death, enables **ErSO-TFPy** to induce massive regression of large breast tumors (500–1500 mm^3^) in mice after a single dose. If recapitulated in humans, such a minimal dosing regimen would revolutionize ERα+ breast cancer therapeutic management through improved treatment compliance, quality-of-life, and long-term outcomes for breast cancer patients.

## Results

### Evaluation of ErSO-TFPy Anticancer Activity and TRPM4-Dependence

**Figure 1:**
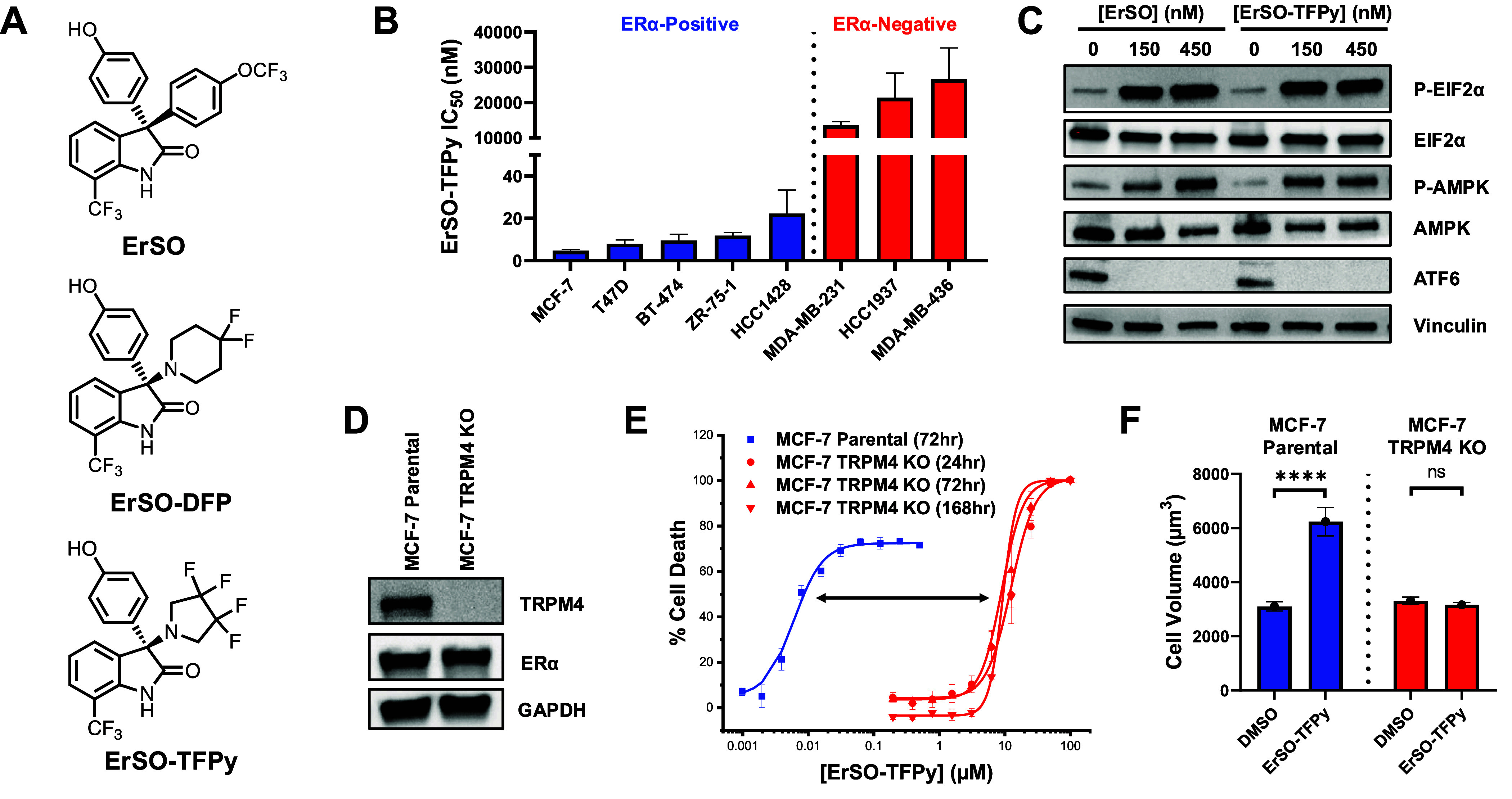
**ErSO-TFPy** anticancer activity is potent and TRPM4-dependent. (A) Chemical structures of **ErSO**, **ErSO-DFP**, and **ErSO-TFPy**. (B) **ErSO-TFPy** IC_50_ values (nM) in a panel of breast cancer cell lines. Cell viability measured via Alamar blue fluorescence at 72 h. (*n* ≥ 2). (C) **ErSO** and **ErSO-TFPy** induce activation of the a-UPR. MCF-7 cells were treated with **ErSO** or **ErSO-TFPy** (0–450 nM) for 6 h. Cells were harvested, lysed, and Western blotted for a-UPR markers (20 μg loaded, *n* = 3). (D) Western blot showing TRPM4 expression (20 μg loaded, *n* = 3). (E) Dose–response curves of **ErSO-TFPy** in MCF-7 parental and MCF-7 TRPM4 KO cells at 24–168 h (*n* ≥ 2). Cell viability measured via Alamar blue fluorescence, Raptinal (100 μM) used as a quantitative dead control. (F) **ErSO-TFPy** induces TRPM4-dependent cell swelling. Cells were treated with vehicle or **ErSO-TFPy** (1 μM) for 2 h following harvesting and measurement of cell diameter (*n* = 3). Statistical significance calculated relative to DMSO using unpaired Student *t* test; **** *P* ≤ 0.0001, ns = not significant.

**ErSO** is a member of the 3-(4-hydroxyphenyl)indoline-2-one class of small molecule anticancer compounds.^21−26^ In an effort to enhance antitumor activity and decrease compound lipophilicity (via nitrogen-incorporation into the scaffold), new **ErSO** derivatives, **ErSO-DFP** and **ErSO-TFPy** (Figure 1A), were designed.^21^**ErSO-TFPy** demonstrated the greatest potency in cell culture, killing sensitive breast cancer cells at single-digit nanomolar concentrations in preliminary studies,^21^ and thus was selected herein for further exploration. In a panel of breast cancer cell lines, **ErSO-TFPy** has potent activity (IC_50_ ≈ 5–25 nM) against multiple ERα-positive cell lines (MCF-7, T47D, BT-474, ZR-75-1, HCC1428) and minimal activity (IC_50_ > 10–30 μM) against ERα-negative breast cancer cell lines (MDA-MB-231, HCC1937, MDA-MB-436) (Figure 1B). In single-dose toxicity experiments using CD-1 mice and Sprague–Dawley rats, **ErSO-TFPy** is well-tolerated, with a maximum tolerated dose (MTD) when given intravenously (IV) of 150 mg/kg in mice, and a rat MTD_IV_ of >50 mg/kg (higher dosages were not assessed), values far superior to the reported MTD of **ErSO** (Figure S1A). Preliminary dosing in research dogs (beagles, using a Kolliphor formulation) also demonstrated the tolerability of **ErSO-TFPy** with an MTD_IV_ of >5 mg/kg (higher doses were not assessed) (Figure S1A). Pharmacokinetic experiments indicate that when given intravenously **ErSO-TFPy** (at 15 mg/kg) reaches concentrations in the blood well above cell culture IC_50_’s for ∼8 h in both mice and rats (Figure S1B). On the basis of **ErSO-TFPy**’s improved potency, cell-line selectivity, and in vivo tolerability, it was advanced to further preclinical evaluation.

Cation dysregulation, hyperactivation of the anticipatory unfolded protein response (a-UPR), and cell-swelling are key features of the cancer cell death induced by this class of compounds.^19,26,27^ Assessment of a-UPR markers demonstrated that, similar to **ErSO**, at nanomolar concentrations **ErSO-TFPy** rapidly induces endoplasmic stress markers in MCF-7 cells (such as cleavage of ATF6, phosphorylation of EIF2α, and phosphorylation of AMPK) (Figure 1C). A recent genome-wide CRIPSR knockout screen revealed TRPM4, a calcium-activated sodium channel present on the plasma membrane, as important to the cell death mechanism induced by **ErSO**, as multiple sensitive cell lines are no longer sensitive to this compound when the gene for TRPM4 is deleted.^20^ To assess the role of TRPM4 in cell death induced by **ErSO-TFPy**, the isogenic cell lines MCF-7 Parental and MCF-7 TRPM4 KO were used (Figure 1D). Knockout of TRPM4 attenuated cell death (∼1000-fold) induced by **ErSO-TFPy**, even at 7-day incubations (Figure 1E). Consistent with this protection, in the MCF-7 parental cell line, **ErSO-TFPy** induced significant cell-swelling with cells approximately doubling in volume; this cell-swelling was not observed in MCF-7 TRPM4 KO cells (Figure 1F).

### ErSO-TFPy Kills Cancer Cells while Other Breast Cancer Clinical Candidates Are Cytostatic

**Figure 2:**
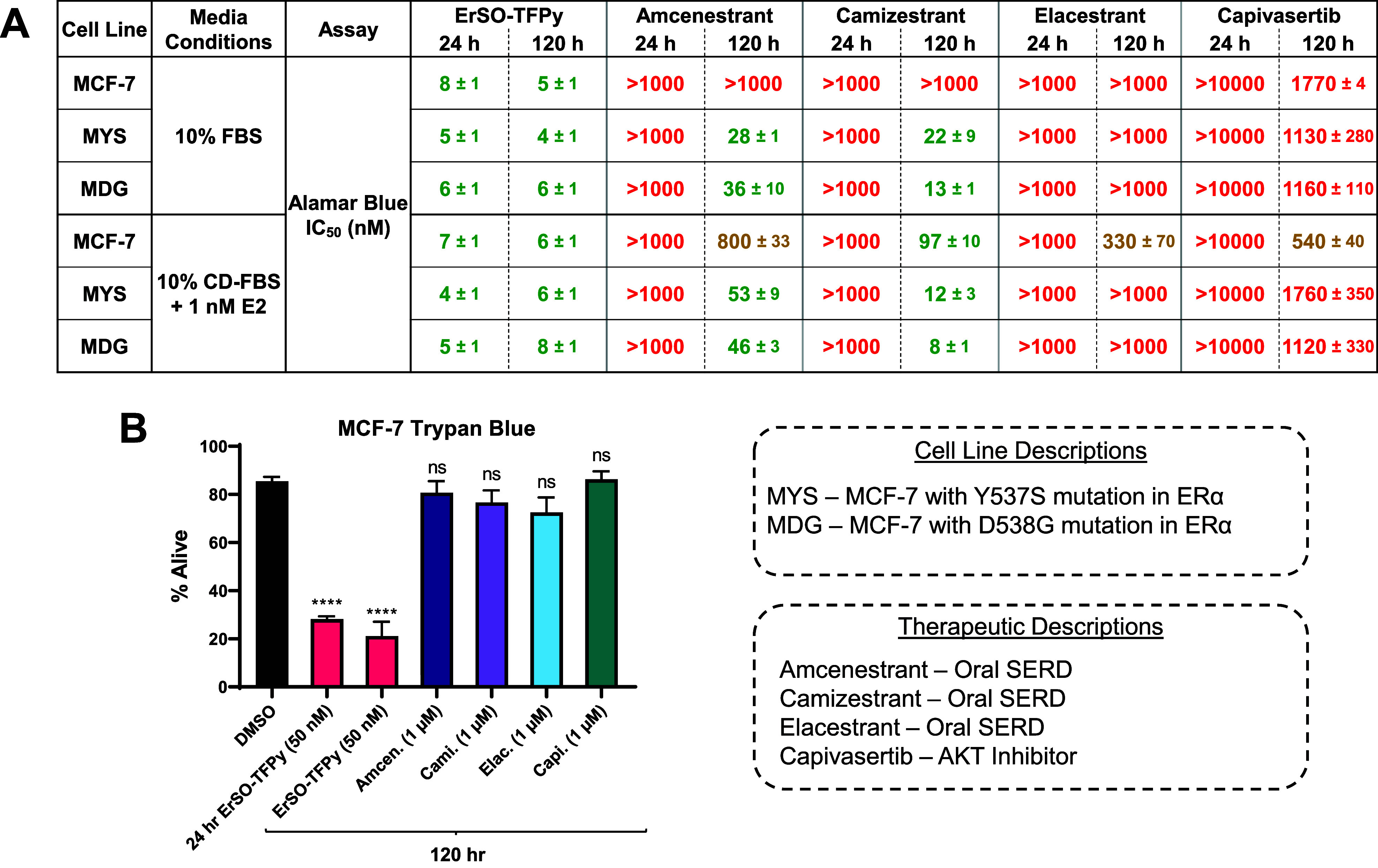
Comparison of **ErSO-TFPy** to clinical drugs for breast cancer. (A) **ErSO-TFPy** was compared with breast cancer drugs in MCF-7 and MCF-7 *ESR1*^mut^ cell lines. CD-FBS = Charcoal stripped FBS. E2 = Estrogen. IC_50_ values calculated by measuring cell viability via Alamar blue fluorescence at 24 or 120 h (*n* ≥ 2), Raptinal (100 μM) used as dead control. IC_50_ values under 100 nM colored green, values between 100 nM and 1000 nM colored yellow gold, and values above 1000 nM colored red. (B) MCF-7 cells treated with compound for 120 h (5 days) (unless noted otherwise) and cell death measured via Trypan blue exclusion assay (*n* = 3). Statistical significance calculated relative to DMSO using unpaired one-way ANOVA; **** *P* ≤ 0.0001, ns = not significant. Descriptions of therapeutics and cell lines provided in boxes.

With the potency, in vivo tolerability, pharmacokinetics, and TRPM4-dependence of **ErSO-TFPy** established, an evaluation was made in a cell culture of how **ErSO-TFPy** compares with other clinically used or emerging drugs for ERα+ breast cancer. Amcenestrant, camizestrant, and elacestrant (recently FDA approved) are orally bioavailable Selective Estrogen Receptor Degraders (SERDs) that have been evaluated in clinical trials.^14,16,28^ Capivasertib is an AKT inhibitor that was approved in combination with fulvestrant for patients with ERα+ breast cancer.^29^**ErSO-TFPy** was assessed alongside these therapeutic agents in cell culture using MCF-7 cells and MCF-7 cells with mutations in *ESR1* (coding variants Y537S and D538G) that lead to estrogen-independent growth of the cells. To limit the effect of unmeasured estrogen levels, SERDs are often evaluated in cell culture using charcoal-dextran-treated fetal bovine serum (CD-FBS) that is then supplemented with estradiol. For this reason, compounds were evaluated using both “stripped” (CD-FBS + 1 nM estradiol) and “unstripped” conditions (FBS, unmeasured estrogens). In the experiment, **ErSO-TFPy** was found to have single-digit nanomolar IC_50_ values regardless of media conditions or *ESR1*-status of MCF-7 cells at both 24 and 120 h time points (Figure 2A, Figure S2). Amcenestrant, camizestrant, and elacestrant all required 120 h (5 days) to exhibit inhibitory effects and were dependent on stripped media for effects against MCF-7 wild type cells, but not MCF-7 mutants (Figure 2A). While elacestrant did have activity against MCF-7 *ESR1* mutant cell lines, it did not reach the 50% inhibition threshold under 1 μM to generate IC_50_ values (Figure S2). Capivasertib exhibited antiproliferative activity at low micromolar concentrations, in line with literature values.^29^

As mentioned, antiestrogen therapeutic interventions typically arrest cell growth leading to a cytostatic effect rather than a cytotoxic effect, which may explain why inhibitory effects in cell culture require 5–7 days.^30^ To assess cell death as opposed to cellular proliferation, MCF-7 cells were treated with compounds at concentrations above the IC_50_ values for up to 5 days and stained with Trypan blue. **ErSO-TFPy** induced significant cell death at both 24 and 120 h, in contrast with the oral SERDs and capivasertib (Figure 2B). These data suggest that the nanomolar IC_50_ values obtained for these clinical breast cancer therapeutics in the Alamar blue viability assay are due to a cytostatic effect and result from inhibiting cell proliferation relative to the proliferating control cells, consistent with previous analyses of this type of therapeutic.^30,31^ In contrast, **ErSO-TFPy** potently kills these breast cancer cells in culture.

### Multiple Doses of ErSO-TFPy Are Highly Efficacious in Murine Tumor Models

**Figure 3:**
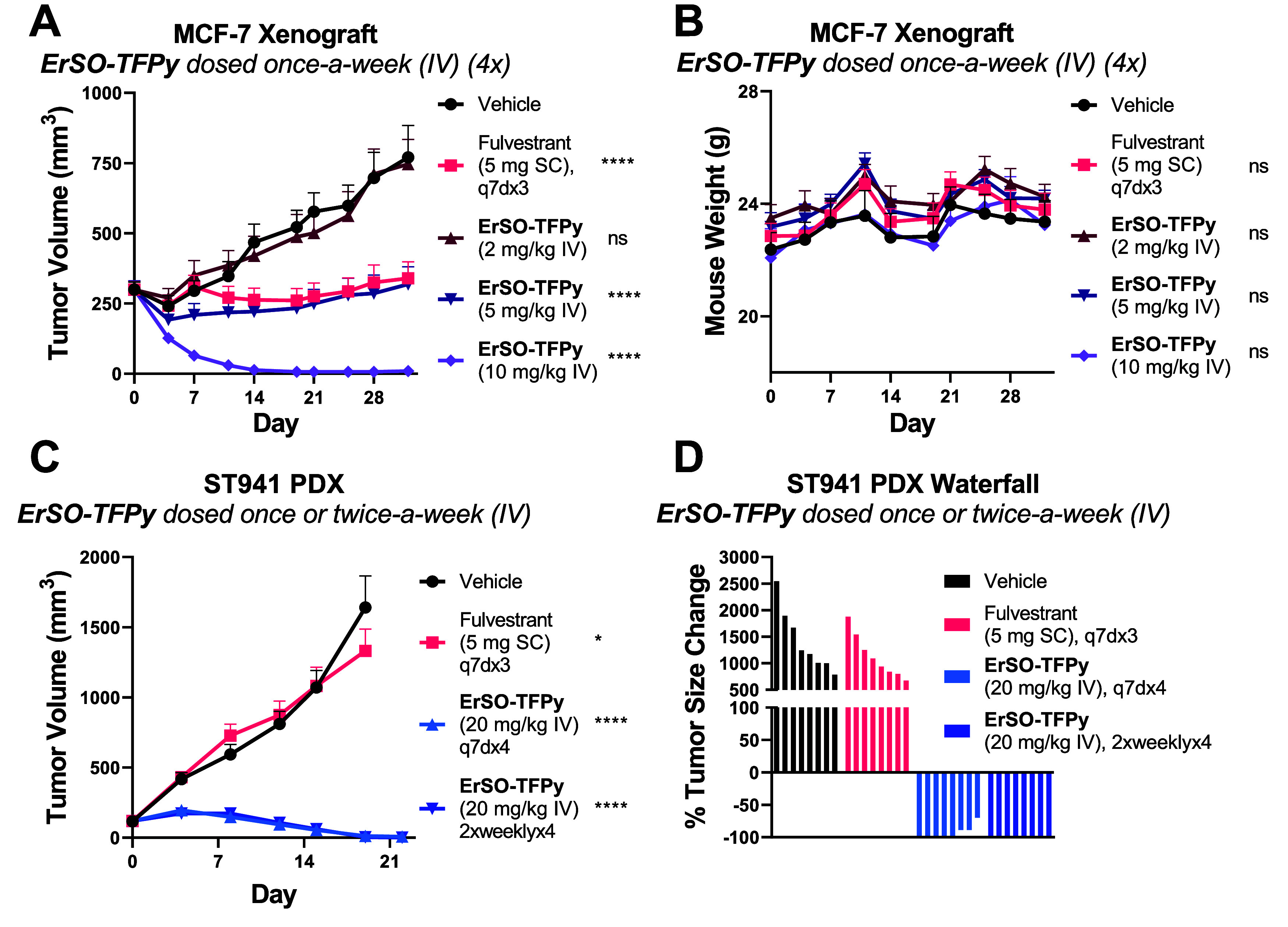
Multiple intravenous doses of **ErSO-TFPy** are efficacious in mouse models. (A) MCF-7 tumors were established in athymic nude mice implanted with an estrogen pellet. When tumors reached an average size of 300 mm^3^, mice were randomly assigned to groups, and treatment began (8 mice/group). Fulvestrant (5 mg/mouse) given subcutaneously (q7dx3). Tumor volume measured over time. (B) Mouse weight over time. (C) ST941 tumors (*ESR1*^*mut*^ (Y537S)) were established in athymic nude mice without estrogen supplementation. Indicated dosing began when tumors reached ∼220 mm^3^ (8 mice/group). Tumor volume measured over time. (D) Percent (%) tumor change at conclusion of study (day 19–21). Both studies were performed by South Texas Accelerated Research Therapeutics. Statistical significance calculated relative to vehicle using two-way ANOVA with Dunnett correction. * *P* ≤ 0.05, **** *P* ≤ 0.0001, ns = not significant.

One of the most promising aspects of **ErSO** is its ability to induce complete tumor regression in preclinical models of breast cancer when given daily or weekly.^19^ This quantitative tumor regression is highly unusual for single-agent breast cancer therapeutics^14−16,28,29,32^ and may be the result of the unique, necrotic mechanism of action for this class of small molecules.^20^**ErSO-TFPy** was tested in a MCF-7 xenograft model in athymic nude mice, using once-a-week dosing (IV, four doses) and compared with fulvestrant (SERD). Clinically, fulvestrant is administered once-a-month as an intramuscular injection, allowing it to achieve stable plasma levels; preclinically, fulvestrant is typically given as a subcutaneous injection, recapitulating clinical efficacy.^33,34^ In this experiment with once-a-week dosing, a dose-dependent effect was observed beginning with significant tumor growth inhibition at 5 mg/kg and complete regression for the group dosed at 10 mg/kg (Figure 3A). This effect is striking, given that 10 mg/kg is well below the MTD_IV_ in mice of 150 mg/kg, indicative of a favorable therapeutic index (TI = 15) for **ErSO-TFPy**. No significant weight loss was observed in any of the treatment groups (Figure 3B).

Patient derived xenografts (PDX) are increasingly preferred in evaluations of preclinical breast cancer therapeutics due to their ability to better recapitulate tumor heterogeneity, behavior, and metastatic potential.^35^**ErSO-TFPy** was evaluated against the drug-resistant ST941 PDX model, derived from a patient previously treated with fulvestrant and bearing *ESR1*^mut^ (encoding Y537S),^14^ implanted in athymic nude mice. Two administration schedules were evaluated, once- and twice-weekly for 4 weeks. Again, even in this very challenging PDX model, complete tumor regressions were observed in 8 of 8 mice when dosed with 20 mg/kg **ErSO-TFPy** twice weekly for 4 weeks (Figure 3C,D). Fulvestrant was ineffective as expected (Figure 3C,D); others have shown that as single agents, even optimized proteolysis targeting chimeras (PROTACs) and SERDs do not induce these types of tumor regressions in this model.^14,32^

### A Single Dose of ErSO-TFPy Is Sufficient for Quantitative Tumor Regressions

**Figure 4:**
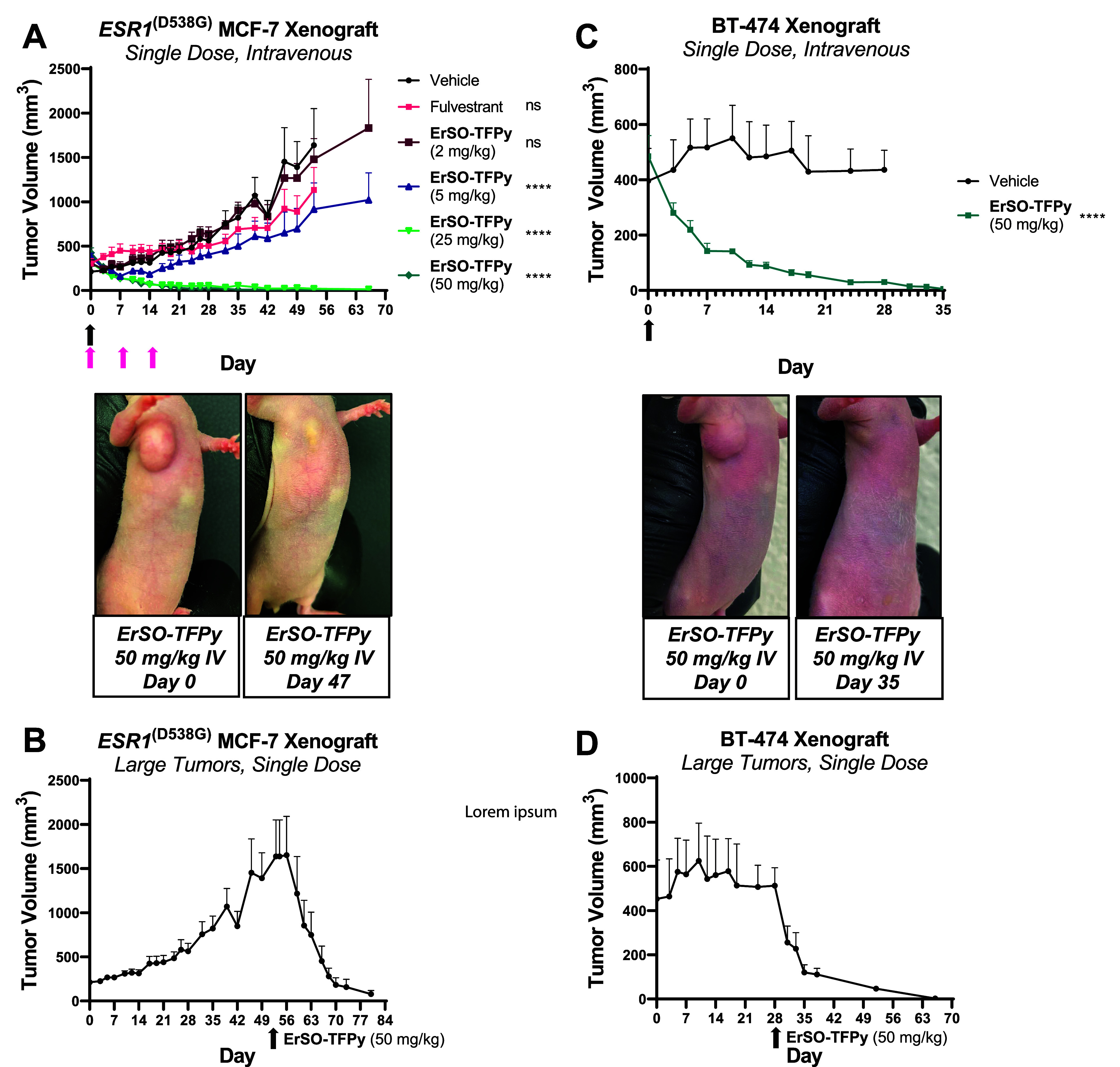
A single dose of **ErSO-TFPy** induces complete tumor regressions in mouse models. (A) MCF-7 *ESR1*^mut^ (D538G) tumors were established in athymic nude mice without estrogen supplementation (≥4 mice/group). Vehicle and **ErSO-TFPy** groups dosed once intravenously at day 0, indicated by the black arrow. Fulvestrant (5 mg/mouse) given subcutaneously (q7dx3), indicated by pink arrows. Tumor volume measured over time. Picture of representative mouse at days 0 and 47 after a single **ErSO-TFPy** (50 mg/kg) treatment. (B) Mice from (A) previously treated with vehicle were given a single dose of **ErSO-TFPy** (50 mg/kg) at day 53. (C) BT-474 tumors were established in athymic nude mice implanted with an estrogen pellet (0.5 mg/pellet, 90-day release). Mice dosed intravenously with vehicle or **ErSO-TFPy** (50 mg/kg) once at day 0 (≥5 mice/group), indicated by black arrow. Pictures of representative mice at days 0 and day 35. (D) Mice with large tumors from (C) previously treated with vehicle (*n* = 4) were given **ErSO-TFPy** (50 mg/kg) at day 28. Statistical significance calculated relative to vehicle using two-way ANOVA with Dunnett correction. **** *P* ≤ 0.0001, ns = not significant.

With the increased tolerability and efficacy at such a low weekly dose of **ErSO-TFPy**, we envisioned the possibility of decreasing the dosing frequency in preclinical tumor models to the point where only a single dose was needed. To make this assessment in the challenging setting of drug-resistant tumors, MCF-7 *ESR1*^mut^ (encoding D538G) cells were implanted in athymic nude mice, and when significant tumors were established (200–300 mm^3^), the animals were treated with a single dose of **ErSO-TFPy** or vehicle at day 0 or weekly with fulvestrant (three doses). At both 25 mg/kg and 50 mg/kg of **ErSO-TFPy**, a single IV dose was sufficient to induce >80% decrease in tumor volume within 14 days; residual tumor masses continued to regress after that point, and 5/5 mice treated with 50 mg/kg **ErSO-TFPy** had no measurable tumors at day 66 (Figure 4A). Following up on this result, an assessment was made to determine if this single treatment of **ErSO-TFPy** would be effective in mice bearing much larger tumors. Mice bearing tumors of ∼1500 mm^3^ (from the vehicle-treated group in Figure 4A) were treated with a single 50 mg/kg dose of **ErSO-TFPy**. Strikingly, even with this large tumor burden, regressions of >90% were observed (Figure 4B). This effect is particularly notable given that typical breast cancer therapeutics require daily dosing for weeks to just achieve retardation of tumor growth in preclinical tumor models.^14,16,28^

To investigate if this effect is generalizable to other breast cancer xenografts models, **ErSO-TFPy** was also evaluated in mice bearing BT-474 (ERα+, HER2+ breast cancer cell line^36^) tumors. When large tumors (∼500 mm^3^) were established in athymic nude mice, mice were treated with a single IV dose of vehicle or **ErSO-TFPy** (50 mg/kg). Once again, BT-474 tumors treated with **ErSO-TFPy** regressed significantly (>80%) over a period of approximately 14 days and continued to regress further over the next weeks (Figure 4C). Consistent with the previous model, when mice bearing larger BT-474 tumors (previously treated with vehicle) were given 50 mg/kg **ErSO-TFPy**, significant tumor regressions were again observed (Figure 4D). An analogous result was observed in a third xenograft model using the HCC1428 cancer cell line (ERα+,*TP53* null^37^) (Figure S3A–C) in NSG (NOD scid gamma) mice. Thus, this single-dose effect occurs in ERα+ cancer cell-line-derived xenografts of varied genetic backgrounds.

### Rapid Induction of Cell Death Results in Single Dose Efficacy

The ability of **ErSO-TFPy** to induce complete regressions after a single dose is surprising given **ErSO-TFPy** serum levels peak within 10 min of administration in mice and are undetectable after 16 h when dosed at 15 mg/kg IV (Figure S1B). The xenograft experiments show that tumor regression occurs over a period of weeks, long after the compound is eliminated. This profound antitumor effect was hypothesized to be a result of either involvement of an immune response even in these immunocompromised mice or due to the rapid cancer cell death induced by **ErSO-TFPy**.

**Figure 5:**
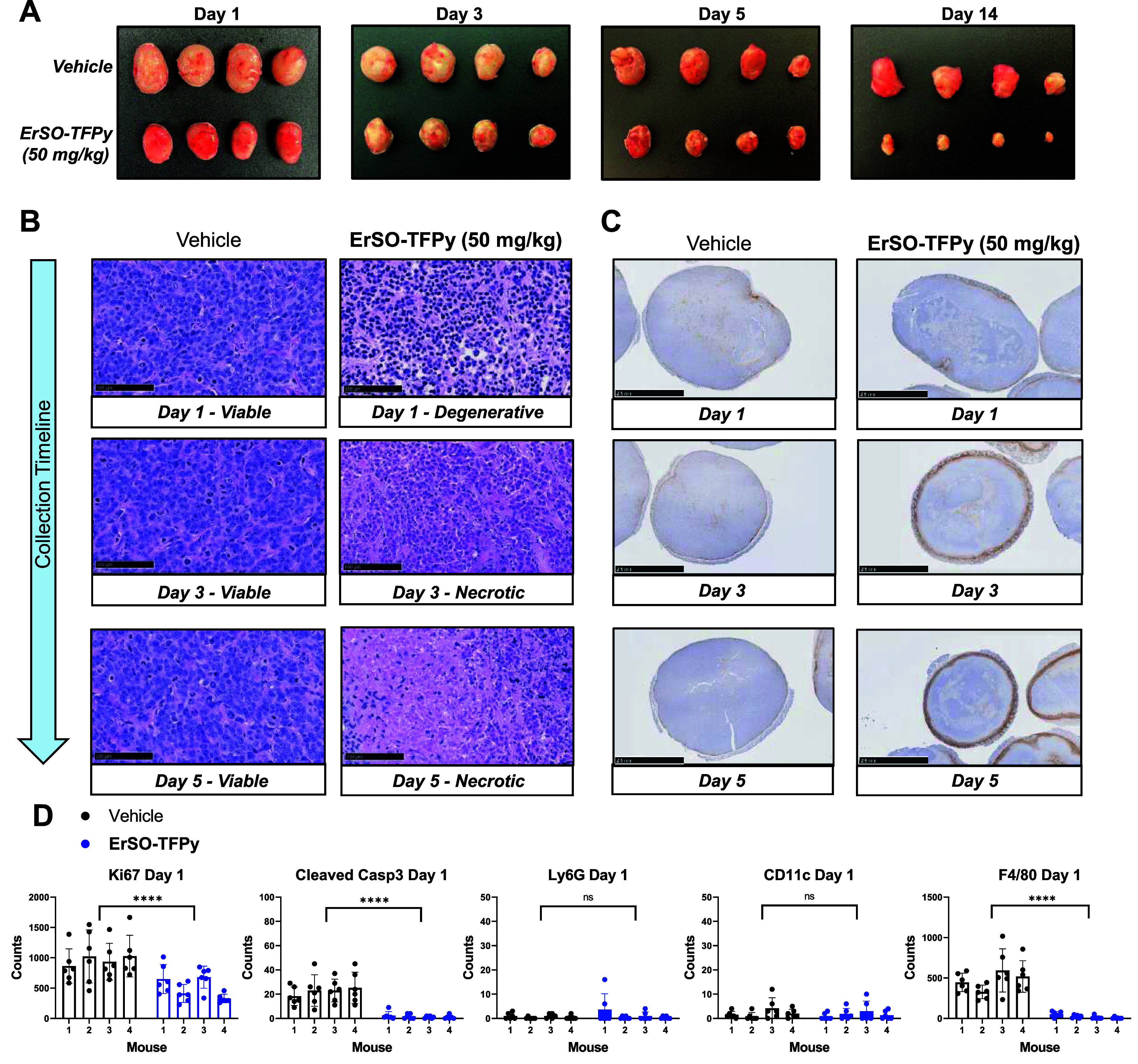
A single dose of **ErSO-TFPy** induces rapid necrosis in tumors, followed by lymphocyte infiltration. (A) MCF-7 *ESR1*^mut^ (D538G) tumors were established in athymic nude mice (4 mice/group). Tumors were collected at indicated time following a single treatment with vehicle or **ErSO-TFPy** (50 mg/kg) intravenously. Average tumor mass for vehicle-treated mice at day 1 was ∼450 mg. (B) Representative H&E-stained regions (days 1, 3, 5). (C) Representative F4/80 images showing macrophage infiltrate over time. (D) ImageJ quantification of Ki67+ (proliferation marker), cleaved Casp3+ (apoptosis marker), Ly6gG+ (neutrophil marker), CD11c+ (dendritic cell marker), and F4/80+ (macrophage marker) populations within viable regions of the tumor at day 1. Statistical significance calculated relative to vehicle using two-way ANOVA with Tukey correction. **** *P* ≤ 0.0001, ns = not significant.

To follow the effect in tumors after the single dose, another group of mice bearing large MCF-7 *ESR1*^mut^ (encoding D538G) tumors were treated with vehicle or a single dose of **ErSO-TFPy** (50 mg/kg) and sacrificed after 1-, 3-, 5-, or 14 days, and tumors were collected for immunohistochemical analysis. Again, a rapid and striking tumor volume reduction was observed in this experiment (Figure 5A, see day 14 tumors). H&E staining of tumors from the vehicle-treated mice showed healthy tumor tissue, outside of a small necrotic core attributed to the fast-growing nature of this tumor. In stark contrast, tumors from mice treated with **ErSO-TFPy** indicate a degenerative state at day 1 and >90% necrotic tissue at 3- and 5-days following treatment (Figure 5B, Figure S4). The data suggest that tumor cell death occurs quickly after treatment with **ErSO-TFPy**, and removal of tumor mass by phagocytic cells (i.e., macrophages) is likely the limiting factor in tumor regression.

Necrosis is often associated with immunogenic cell death, as necrotic cells typically release ATP and Damage Associated Molecular Patterns (DAMP’s) that recruit immune cells.^38^ In this xenograft model, where athymic nude mice were used, these animals lack T cells but retain other myeloid-derived immunocytes (macrophages, dendritic cells, neutrophils) that could play a role in the **ErSO-TFPy** induced tumor regression. However, staining for neutrophils (Ly6G), dendritic cells (CD11c), and macrophages (F4/80) at day 1 indicated very few infiltrating neutrophils or dendritic cells in both vehicle and **ErSO-TFPy** treated tumors (Figure 5C,D, Figure S4). Macrophages were decreased in the viable regions of **ErSO-TFPy** treated tumors (Figure 5D), most likely due to recruitment to necrotic areas for phagocytosis. At days 3 and 5, staining indicated increased infiltration of macrophages and neutrophils in **ErSO-TFPy** treated tumors (Figure 5C, Figure S4). These data indicate that immune cells are likely involved in the phagocytosis of necrotic tumor tissue, but in this model, which lacks T cells, there is minimal involvement of the immune populations assessed in the induction of the antitumor effect of **ErSO-TFPy**. However, there is a possibility that macrophage and neutrophil infiltration at later days (3 and 5) is responsible for elimination of a small number of residual, viable tumor cells.

Further analysis was focused on the viable regions within treated tumors collected at day 1. Ki67 staining (proliferative marker) was decreased in **ErSO-TFPy** treated tumors but remained significant (Figure 5D), indicating Ki67-independent cell death. Basal levels of cleaved caspase 3 (marker of apoptosis, cleaved Casp3+) seen in vehicle-treated tumors were decreased upon treatment with **ErSO-TFPy** (Figure 5D). In combination with H&E staining, the data support necrosis as the mechanism of cell death.

Follow-up studies in cell culture indicate that **ErSO-TFPy** kills MCF-7 cells more quickly than **ErSO** and **ErSO-DFP** (Figure S5A). In sensitive cells, even very short incubations (2 h) are enough to initiate cell death (Figure S5B). These data, in combination with the degenerative phenotype seen at day 1 in the IHC studies (Figure 5B), suggest that the profound antitumor activity of **ErSO-TFPy** is largely due to the rapid induction of cancer cell necrotic death rather than mediated by activation of the immune system in these immune-deficient mice.

## Discussion

The vast majority of small molecule anticancer drugs are administered via multiple and frequent dosing schedules. In preclinical models, it is common to evaluate the anticancer activity of a drug using tumor volumes of ∼100–200 mm^3^.^39^ Even when treating these small tumors in mice, examples of small molecule drugs that induce complete or near-complete tumor regressions are rare and require multiple doses.^40,41^ Some immunotherapies like chimeric antigen receptor T cells (CAR-T) have efficacy following a single infusion of CAR-positive T cells (KYMRIAH, YESCARTA, ABECMA); of course these complex therapies have high associated costs and have yet to show strong efficacy in ERα+ breast cancer.^42^

An anticancer regimen that consists of a single dose, or a handful of doses, could change the face of breast cancer treatment.^7^ While endocrine therapy is a major advance in the management of this disease,^43^ adjuvant endocrine therapy requires patients to take daily medications for up to a decade, including for leading drugs tamoxifen (20 mg/day), letrozole (2.5 mg/day), and anastrazole (1 mg/day). The reduced compliance^10,11^ to these regimens is a direct result of the physical and financial toxicities^44^ that decrease patient quality of life and plays a role in the ineffectiveness of long-term endocrine therapy. Given the continued challenge in treatment of ERα+ breast cancer (especially in the advanced, drug-resistant setting), many therapies targeting ERα are being developed and investigated.^45^ However, in preclinical models, even optimized SERDs and PROTACs targeting ERα are largely cytostatic and fail to induce dramatic tumor regression when used as single agents.^14−16,28,32^ When used in combination with inhibitors of CDK4/6, PI3K, or AKT some regression may be achieved in moderately sized tumors (100–300 mm^3^),^14−16,32^ but complete tumor eradication is not observed.

**ErSO-TFPy** rapidly kills ERα+ breast cancer cells at low nanomolar concentrations in essentially a quantitative fashion and is well-tolerated in multiple species (mice, rats, and dogs). Surprisingly given its relatively short half-life in vivo (SI Figure 1), a single IV dose of **ErSO-TFPy** is sufficient to induce complete or near-complete tumor regressions in three different human tumor models of breast cancer in immunocompromised mice. This is a consistent observation with a variety of tumor sizes, most impressively with ∼1000 mm^3^ tumors regressing >80%. The data demonstrate that **ErSO-TFPy** is a highly effective and well-tolerated single-dose antitumor agent. These studies also provide an unusual case study of pharmacokinetics, where the short exposure times in the blood of mice do not limit **ErSO-TFPy** from initiating tumor cell death, likely due to the nanomolar potency and rapid necrotic phenotype induced by **ErSO-TFPy**. Currently, these efficacy studies are limited to immune deficient mice (NSG and athymic nude), and the immune components that are present (macrophages, dendritic cells, neutrophils) do not appear to be involved in tumor cell death. Advancing this compound as a therapeutic will require additional toxicology studies in rodents and dogs; given the induction of tumor cell necrosis, it is possible that **ErSO-TFPy** could also activate the immune system in patients, potentially increasing efficacy and limiting resistance. TRPM4 expression is upregulated in breast cancer and is associated with an estrogen response,^46,47^ increased migration of cancer cells,^48,49^ and a-UPR proteins are strongly overexpressed in ERα-positive breast cancers.^50^ Given the overexpression of TRPM4 in other cancers^49,51^ such as colorectal^52^ and prostate,^53^ investigation of **ErSO-TFPy** in these and other cancers is warranted.

The tumor regressions in diverse models reported herein showcase the value of compounds that rapidly kill cancer cells. For routine assessment of drug candidates against cancer cell lines in culture, cell death (or cell growth inhibition) is typically measured after longer incubations, often from 3 to 7 days. The observations reported here, of **ErSO-TFPy** inducing rapid death of cancer cells in culture and also profound tumor regressions in vivo, suggest that evaluating putative anticancer compounds at shorter incubation times (<1 day) could have merit. The ability of **ErSO-TFPy** to induce rapid and selective death in cell culture leading to complete tumor regressions in mice after a single dose highlights the potential of therapeutics that induce rapid death of cancer cells, and if translated to humans, this compound would provide a significant clinical benefit.

## Materials and Methods

### Cell Lines and Culturing Conditions

All cell lines were cultured at 37 °C with 5% CO_2_. All cells were grown in medium lacking phenol-red. MCF-7 cells were grown in Eagles Minimum Essential Media (EMEM) supplemented with 10% Fetal Bovine Serum (FBS) and 1% Penicillin-Streptomycin (P/S). T47D cells were grown in Minimum Essential Media (MEM) supplemented with 10% FBS and 1% P/S. BT-474, Zr-75-1, HCC1428, MDA-MB-231, and HCC1937 cells were grown in RPMI 1640 supplemented with 10% FBS and 1% P/S. MDA-MB-436, MYS, and MDG cells were grown in Dulbecco’s Minimum Essential Media (DMEM) supplemented with 10% FBS and 1% P/S. MCF7-Parental and MCF7-TRPM4 KO cells were provided by Professor David Shapiro’s laboratory (University of Illinois at Urbana-Champaign) and cultured using MEM supplemented with 10% FBS and 1% P/S. All cell lines were used directly from ATCC stocks and/or submitted to University of Arizona Genetics Core (UAGC) or IDEXX for authentication via STR profiling.

### Alamar Blue Fluorescence for Cell Viability (IC_50_)

6,000–10,000 cells were seeded per well in 99 μL of appropriate media in 96-well plates and allowed to adhere overnight. One μL of compound-containing DMSO solution was added to each well to give a final volume of 100 μL (DMSO final concentration = 1%). Compounds were incubated for 24 h-168 h before aspirating media and replacing with fresh media (100 μL). Ten μL of Alamar blue solution (1 mg of resazurin dissolved in 10 mL of PBS) was added to each well. After 4–6 h of incubation, fluorescence (λexcitation = 555 nm, λemission = 585 nm) was measured using a SpectraMax M3 plate reader (Molecular Devices). Five technical replicates per concentration. Percent dead was calculated using 100 μM Raptinal as a 100% dead control. Dose response curves and IC_50_ values were calculated using Origin Pro V10.

### Immunoblotting/Western Blot Procedure

Cells were seeded in 6-well plates and allowed to adhere overnight (∼18 h). After the indicated treatment, cells were lysed using RIPA buffer containing phosphatase inhibitor (BioVision, 1:50 dilution) and protease inhibitor (Calbiochem, 1:100 dilution). Protein concentrations were determined using a BCA assay (Pierce). Loading dye (BIO-RAD) w/beta-mercaptoethanol was added to lysate, and samples were boiled at 95 °C. 10–20 μg of protein was loaded on to 4–20% polyacrylamide gels (BioRad), resolved by SDS-PAGE, and then transferred onto a membrane (PDVF Millipore) for antibody staining. Blots were blocked with BSA solution (2 g in 40 mL of TBST) for 1 h. Primary antibody was added and incubated overnight with rotation at 4 °C. Blots were then washed with Tris-Buffered Saline + Tween-20 (TBST), and secondary antibody was added and incubated for 1 h. Following washing, blots were incubated with SuperSignal West Pico Plus according to manufacturer’s instructions and imaged on a BioRad GelDoc. Antibodies were purchased from Cell Signaling unless noted otherwise. Note: For TRPM4 (#CF50038, Origene) protein lysates were not boiled at 95 °C following loading dye (+beta-mercaptoethanol) addition; instead, they were heated at 37 °C for 45–60 min.

### Cell Swelling

MCF-7 Parental or MCF-7 TRPM4 KO cells were plated in a 6-well plate at 300,000 cells/well. The next day, the cells were treated by vehicle or 1 μM **ErSO-TFPy** for 2 h. Then, the cells were harvested and centrifuged at 500 rpm for 5 min. After resuspending the cells in 100 μL of fresh medium, 10 μL of the sample was loaded in a slide and imaged with an automatic cell counter, Countess II (Thermo Fisher). The cell diameter was then automatically obtained from the cell counter. Spherical volume assumed, *V* = 4/3(π)r^3^.

### MCF-7 Xenograft

Study conducted by South Texas Accelerated Research Therapeutics (START #111-22123-MCF-7). For full experimental details, please contact START. In brief, 10 × 10^6^ MCF-7 cells were implanted in the flank of athymic nude mice (with exogenous estradiol). Dosing began when tumors reached an average size of 220 mm^3^ (8 mice/group). **ErSO-TFPy** formulated in 2.5% Ethanol, 5% Kolliphor EL, 15% Propylene Glycol, and 77.5% Sterile saline and dosed weekly, intravenously (4x). Fulvestrant formulated in 10% ethanol, 90% castor oil and dosed weekly, subcutaneously (3×).

### ST941 Xenograft

Study conducted by South Texas Accelerated Research Therapeutics (START #111-22180-ST941). For full experimental details, please contact START. In brief, athymic nude mice were implanted on the flank with tumor fragments from host animals (8 mice/group). **ErSO-TFPy** formulated in 2.5% Ethanol, 5% Kolliphor EL, 15% Propylene Glycol, 77.5% Sterile saline and dosed weekly, intravenously (4×). Fulvestrant was formulated in 10% ethanol and 90% castor oil and dosed weekly, subcutaneously (4×).

### MCF-7 ESR1^mut^ (encoding D538G variant) Xenograft

Athymic nude mice (female) were implanted with 1.5 × 10^6^ MCF-7 *ESR1*^mut^ (D538G) cells in a mixture of Hanks Balanced Salt Solution (HBSS)/Matrigel (1:1) in the mammary fat pad. Tumors were treated with **ErSO-TFPy** or vehicle intravenously on day 0 or weekly (3×) with fulvestrant (5 mg) subcutaneously (≥4 mice/group). **ErSO-TFPy** was formulated in 2.5% Ethanol, 5% Kolliphor EL, 15% Propylene Glycol, and 77.5% Sterile saline. Fulvestrant formulated in castor oil. Tumor volume measured via caliper. IACUC protocol #23020.

### Immunohistochemistry

Athymic nude mice (female) were implanted with 1.5 × 10^6^ MCF-7 ESR1^mut^ (D538G) cells. At the beginning of treatment, mice were randomized between treatments (mice with larger tumors were placed in groups with earlier collection dates). Mice were treated with vehicle or 50 mg/kg **ErSO-TFPy** intravenously (4 mice/group). Tumors were collected at the indicated time point. Tumors were stored in 10% formalin and shipped to the UChicago HTRC for immunohistochemistry. Samples were embedded in paraffin, sectioned (5 μM thickness), and stained with antibody as indicated using a Leica Bond RX Automatic Scanner. Slides were imaged using a Nanozoomer Slide Scanner. For day 1 quantifications, 6 images were taken at 20× magnification and quantified using ImageJ. Antibody Information: CDIIc (#97585S, Cell Signaling), Cleaved Caspase-3 (#9661, Cell Signaling), F4 80 (#MCA497GA, AbD Serotec), Ki67 (#RM-9106-s, Thermo Scientific Labvision), and Ly6G (#127602, Labvision). IACUC protocol #23020.

## Supporting Information Available

Supplemental figures and materials/methods including tolerability/pharmacokinetic data, dose–response curves, full immunohistochemistry images, and cell death kinetic experiments (PDF)
