# Supplementary material for: Single Dose of a Small Molecule Leads to Complete Regressions of Large Breast Tumors in Mice
Source: ACS Cent Sci. 2025 Jan 22;11(2):228–38. doi: 10.1021/acscentsci.4c01628 (PMC11869136; doi:10.1021/acscentsci.4c01628)
Supplement: Supplementary file 1 — oc4c01628_si_001.pdf [file oc4c01628_si_001.pdf]

Supplemental Information For:

Single Dose of a Small Molecule Leads to Complete Regressions of Large Breast Tumors in Mice

Michael P. Mulligan,[1,2] Matthew W. Boudreau,[1,2] Brooke A. Bouwens[2,3], Yoongyeong Lee[4], Hunter W. Carrell,[1,2] Junyao Zhu,[3] Spyro Mousses,[5] David J. Shapiro,[3,6] Erik R. Nelson,[2,6,7,8,9] Timothy M. Fan,[2,6,10] Paul J. Hergenrother[1,2,6*]

[1]Department of Chemistry, University of Illinois at Urbana-Champaign, Urbana, IL 61801, USA
[2]Carl R. Woese Institute for Genomic Biology University of Illinois at Urbana-Champaign, Urbana, IL 61801, USA
[3]Department of Biochemistry, University of Illinois at Urbana-Champaign, Urbana, IL 61801, USA
[4]Department of Comparative Biosciences, University of Illinois at Urbana-Champaign, Urbana, IL 61802, USA
[5]Systems Oncology, Scottsdale, AZ 85255, USA
[6]Cancer Center at Illinois, University of Illinois at Urbana-Champaign, Urbana, IL 61801, USA
[7]Department of Molecular and Integrative Physiology, University of Illinois at Urbana-Champaign, Urbana, IL 61801, USA
[8]Division of Nutritional Sciences, University of Illinois at Urbana-Champaign, Urbana, IL, USA
[9]Beckman Institute for Advanced Science and Technology, University of Illinois at Urbana-Champaign, Urbana, IL 61801, USA
[10]Department of Veterinary Clinical Medicine, University of Illinois at Urbana-Champaign, Urbana, IL 61802, USA

*Correspondence: hergenro@illinois.edu (PJH)

Table of Contents

Supplemental Fig. 1 Tolerability and pharmacokinetic experiments 3

Supplemental Fig. 2 Dose response curves for comparison with clinical candidates. 4

Supplemental Fig 3. Single dose ErSO-TFPy induces regression of HCC1428 tumors. 5

Supplemental Fig. 4 Full images from immunohistochemistry study. 6

Supplemental Fig. 5 ErSO-TFPy kills rapidly in cell culture. 7

Materials and Methods 8

A

MTD Summary

Organism	Administration	**ErSO**	**ErSO-DFP**	**ErSO-TFPy**
Mouse	PO	>150 mg/kg[a]	>200 mg/kg[a]	>200 mg/kg
	IV	20 mg/kg[a]	95 mg/kg[a]	150 mg/kg
Rat	IV	10 mg/kg[a]	>50 mg/kg[a]	>50 mg/kg
Dog	IV	n.d.	n.d.	>5 mg/kg

B

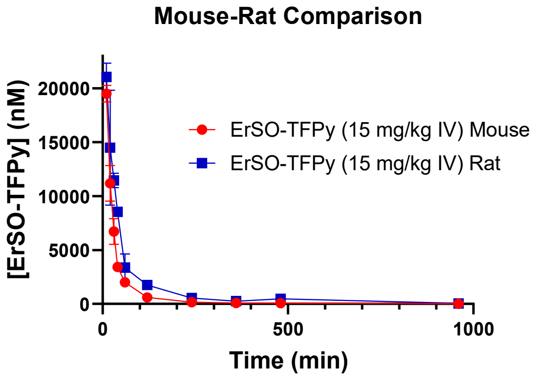

**Parameters**	**ErSO-TFPy (IV, 15 mg/kg)**	
	**Mouse**	**Rat**
**C_{max} (ng/mL)**	15,800	23,000
**AUC (h*ng/mL)**	5,400	9,900
**$t_{1/2}$ (h)**	1.5	2.6
**MRT (h)**	0.77	1.4
**CL (ml/hr/kg)**	2,780	1,560

Supplemental Figure 1. Tolerability and pharmacokinetic experiments.

(**A**) Results of single-dose tolerability experiments in CD-1 mice, Sprague Dawley Rats, and Beagles. MTD = Maximum Tolerated Dose. PO = Oral administration. IV = Intravenous Administration. Mouse and Rat tolerability assessed using formulation A: 5% DMSO, 10% Tween, 85% PBS. Dog tolerability collected using Formulation B: 2.5% Ethanol, 5% Kolliphor EL, 15% Propylene Glycol, 77.5% Sterile saline. [a] reported by Boudreau, M.W.; *et al. J. Med. Chem.* **2022**, *65,* 3894. (**B**) PK results following 15 mg/kg (IV) dose in Balb/c mice and Sprague Dawley rats (n=3). Formulation B used. AUC = Area Under Curve. MRT = Median Residence Time. CL = Clearance.

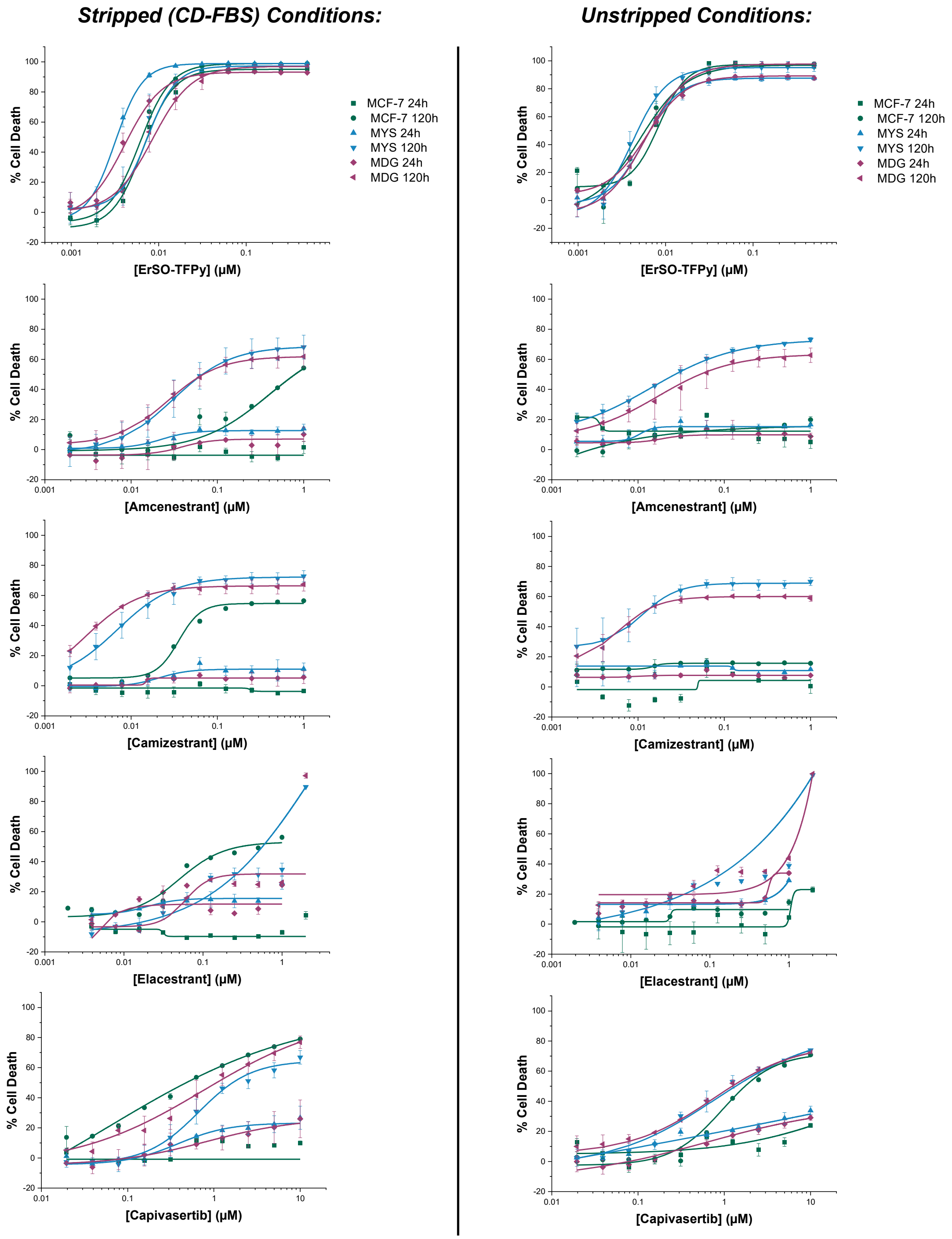

Supplemental Figure 2. Dose response curves for comparison with clinical candidates.

Results from cell culture experiments evaluating **ErSO-TFPy** against clinical candidates for breast cancer in MCF-7, MYS, and MDG cells using alamar blue fluorescence at 24/120 hours (n ≥ 2), 100 uM Raptinal used as dead control. Stripped = 10% CD-FBS supplemented with 1 nM estradiol. Unstripped = 10% FBS (unmeasured levels of estrogens). Curves generated using OriginPro V10.

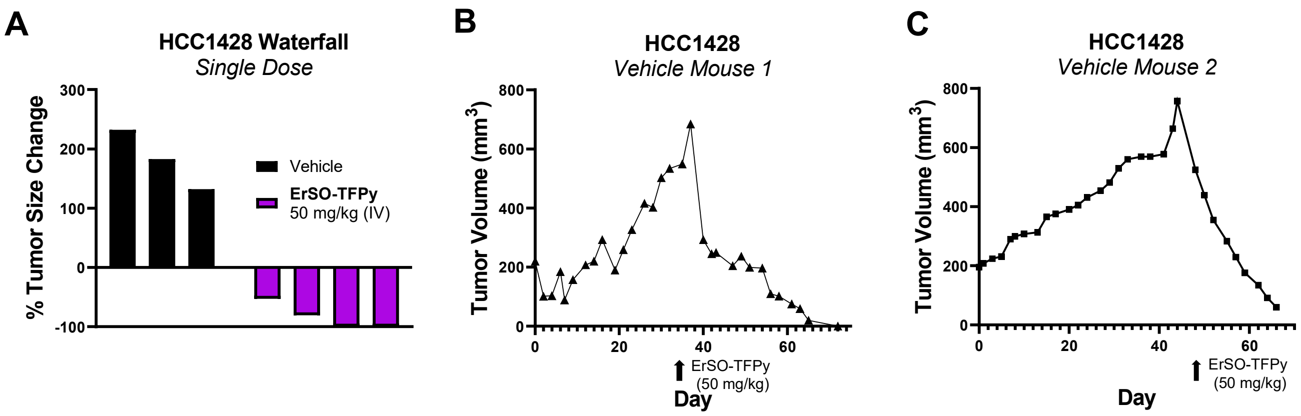

Supplemental Figure 3. Single dose ErSO-TFPy induces regression of HCC1428 tumors.

(**A**) Waterfall plot summarizing results from HCC1428 at day 28 following single dose of vehicle (n=3) or 50 mg/kg IV **ErSO-TFPy** (n=4). Female NSG mice were implanted with estrogen pellet (0.72 mg/pellet, 60-day release). Treatment began when tumors were 200-500 mm^3. (**B**) Individual mouse with large tumor (previously treated with vehicle) was treated with a single dose of 50 mg/kg IV **ErSO-TFPy** at day 37. (**C**) Individual mouse with large tumor (previously treated with vehicle) was treated with a single dose of 50 mg/kg IV **ErSO-TFPy** at day 44.

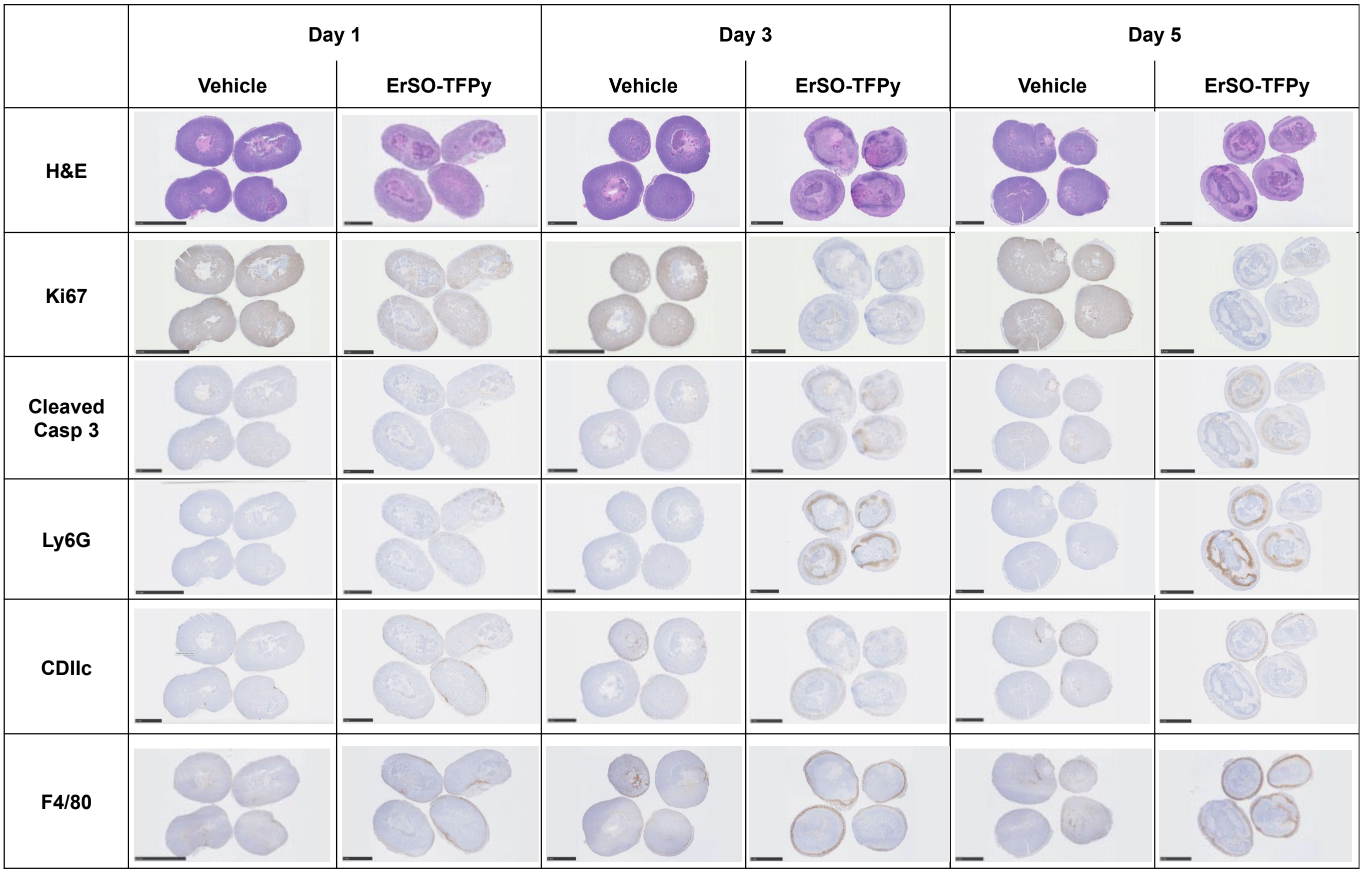

Supplemental Figure 4. Full images from immunohistochemistry study.

MCF-7 *ESR1*^{mut} (encoding D538G variant) tumors were established in athymic nude mice (4 mice/group). Tumors were collected at indicated time following a single treatment with vehicle or **ErSO-TFPy** (50 mg/kg) intravenously. Tumors were shipped to UChicago HTRC for immunohistochemistry using indicated markers and slides were imaged using Nanozoomer Slide Scanner. H&E = Hematoxylin and eosin. Ki67 = Marker for proliferation. Cleaved Casp 3 = Marker for apoptosis. Ly6G = Marker for neutrophils. CDIIc = Marker for dendritic cells. F4/80 = Marker for macrophages.

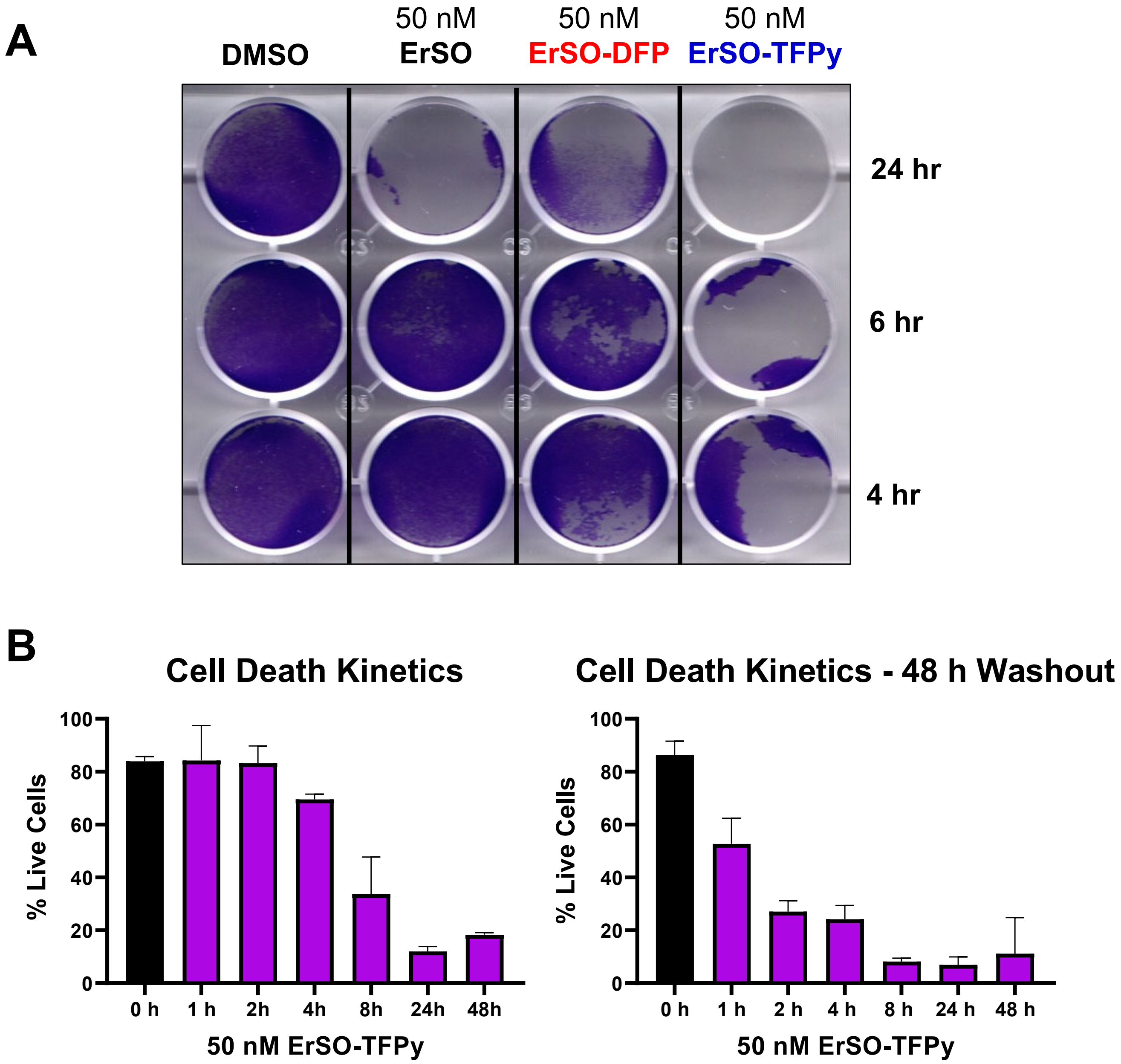

Supplemental Figure 5. ErSO-TFPy kills sensitive cancer cells rapidly in cell culture.

(**A**) Crystal violet staining of MCF-7 cells following treatment with indicated compounds from 4-24 hr (n=2). (**B**) Left – Assessment of MCF-7 cell death via trypan blue following treatment with **ErSO-TFPy** (n=2). Right – Washout experiment. MCF-7 cells treated with **ErSO-TFPy** for indicated time and cell death was assessed via trypan blue at 48 hours after beginning treatment (n=2).

Materials and Methods

Tolerability Experiments

For mice and rat tolerability, **ErSO-TFPy** was formulated in 5% DMSO, 10% Tween-20, 85% PBS and administered orally or intravenously to female CD-1 mice (Charles River) and Sprague Dawley rats (Charles River). If a given dose was tolerated, higher doses were administered until an intolerable dose was observed or solubility limit was reached. Rodents were monitored for signs of distress, lethargy, and neurotoxicity. MTD defined as maximum dose in which acute side effects were tolerated and no lethality was observed.

For dog tolerability, **ErSO-TFPy** was formulated in 2.5% Ethanol, 5% Kolliphor EL, 15% Propylene Glycol, 77.5% Sterile saline and administered via intravenous infusion over 30 minutes to female beagle dogs. Dogs were monitored for physical reactions, weight loss, food intake, and clinical chemistry/hematology panels were performed.

Pharmacokinetic Experiments

Pharmacokinetic experiments were conducted by Translational Drug Development (TD2).
Mouse study: 5-8 week old female Balb/c mice were used. **ErSO-TFPy** was formulated in 2.5% Ethanol, 5% Kolliphor EL, 15% Propylene Glycol, 77.5% Sterile saline and 15 mg/kg administered intravenously. Mice were sacrificed and blood collected at predetermined time points: 10 min, 20 min, 40 min, 1 hr, 2 hr, 4 hr, 8 hr, 16 hr (n=3). Blood placed in Li-Heparin tubes and centrifuged at 4°C to collect plasma. Plasma transferred to cryovial, snap-frozen with liquid nitrogen and stored at -80°C. Concentration of **ErSO-TFPy** in plasma was determined by LC-MS/MS analysis at Duke Department of Biostatistics and Bioinformatics.
Rat Study: Female Sprague Dawley rats used. ErSO-TFPy was formulated in 2.5% Ethanol, 5% Kolliphor EL, 15% Propylene Glycol, 77.5% Sterile saline and 15 mg/kg administered intravenously. Blood was collected at predetermined time points: 10 min, 20 min, 40 min, 1 hr, 2 hr, 4 hr, 8 hr, 16 hr (n=3). Blood placed in Li-Heparin tubes and centrifuged at 4°C to collect plasma. Plasma transferred to cryovial, snap-frozen with liquid nitrogen and stored at -80°C. Concentration of **ErSO-TFPy** in plasma was determined by LC-MS/MS analysis at Duke Department of Biostatistics and Bioinformatics.

Alamar Blue Fluorescence for Cell Viability (IC_{50}) with CD-FBS (Stripped)

Breast cancer therapeutics were purchased from MedChemExpress: Amcenestrant (#HY-133017), Camizestrant (#HY-136255), Elacestrant (#HY-19822), Capivasertib (#HY-15431). MCF-7 cells were cultured using EMEM supplemented with 10% CD-FBS, 1% P/S, and 1 nM estradiol (MedChemExpress: # HY-B0141). MYS and MDG cells were cultured using DMEM supplemented with 10% CD-FBS, 1% P/S, and 1 nM estradiol. 6,000-10,000 cells were seeded per well in 99 µL of appropriate media in 96-well plates and allowed to adhere overnight. 1 µL of

compound-containing DMSO solution was added to each well to give a final volume of 100 µL (DMSO final concentration = 1%). Compounds were incubated for 24 hours or 120 hours before aspirating media and replacing with fresh media (100 µL). 10 µL of Alamar blue solution (1 mg resazurin dissolved in 10 mL of PBS) was added to each well. After 4-6 hours of incubation, fluorescence (λexcitation = 555 nm, λemission = 585 nm) was measured using a SpectraMax M3 plate reader (Molecular Devices). 5 technical replicates per concentration. Percent dead was calculated using 100 µM Raptinal as a 100% dead control. Dose response curves and IC_{50} values were calculated using Origin Pro V10.

BT474 Xenograft

Athymic nude mice (female) bearing estrogen pellets (Innovative Research of America, 0.5 mg/pellet, 90-day release) were implanted with 10 x 10^6 BT474 cells in a mixture of HBSS/Matrigel (1:1) in the mammary fat pad. Tumors were treated with 50 mg/kg **ErSO-TFPy** or vehicle intravenously (≥ 5 mice/group). **ErSO-TFPy** formulated in 2.5% Ethanol, 5% Kolliphor EL, 15% Propylene Glycol, 77.5% Sterile saline. Tumor volume measured via caliper. IACUC protocol #23020.

HCC1428 Xenograft

NSG mice (female) bearing estrogen pellets (Innovative Research of America, 0.72 mg/pellet, 60-day release) were implanted with 1 x 10^6 HCC1428 cells in a mixture of DMEM/Matrigel (1:1). Tumors were treated with 50 mg/kg **ErSO-TFPy** or vehicle intravenously (≥ 3 mice/group). **ErSO-TFPy** formulated in 2.5% Ethanol, 5% Kolliphor EL, 15% Propylene Glycol, 77.5% Sterile saline. Tumor volume measured via caliper. IACUC protocol #23020.

Crystal Violet

Cells were seeded in 12-well plates and allowed to adhere overnight. Following compound incubation, media was removed, and cells were washed with PBS. 1 mL crystal violet solution (250 mg crystal violet in 40 mL H_2O, then 10 mL MeOH) was added and plate was rocked at room temperature for 20 minutes. Crystal violet solution was aspirated followed by 5 washes with tap water and plate was imaged using high resolution scanner.

Trypan Blue Exclusion Assay

300,000 MCF-7 were seeded 6/12-well plates and allowed to adhere overnight. Compounds were incubated for indicated time. Media removed, followed by PBS wash, and trypsinization (collected all). Cells were spun down at 300g for 3 minutes and resuspended in 1 mL media. Cells mixed with trypan blue stain (0.4%, Gibco) and live/dead counted via hemacytometer. For comparison with breast cancer clinical therapeutics, MCF-7 were cultured using EMEM w/ 10% CD-FBS + 1nM estradiol. For washout experiments, MCF-7 were incubated with **ErSO-TFPy** for indicated

time, then media was removed and replaced with fresh media and cell death assessed at 48 hours from start of experiment.
